# Exploring Post-treatment Weight Changes in Overweight and Obese Patients With Graves’ Disease: A Retrospective Analysis

**DOI:** 10.7759/cureus.59408

**Published:** 2024-04-30

**Authors:** Maria Leonor Guia Lopes, Carlos Bello, José Pedro Cidade, Clara Cunha, Clotilde Limbert, Joao Sequeira Duarte

**Affiliations:** 1 Endocrinology, Centro Hospitalar Lisboa Ocidental - Hospital Egas Moniz, Lisbon, PRT; 2 Endocrinology, Hospital da Luz Lisboa, Lisbon, PRT; 3 Intensive Care Medicine, Centro Hospitalar Lisboa Ocidental - Hospital São Francisco Xavier, Lisbon, PRT; 4 Clinical Medicine, NOVA Medical School - Universidade NOVA de Lisboa, Lisbon, PRT

**Keywords:** hyperthyroidism, cardiovascular risk, obesity, weight gain, graves’ disease

## Abstract

Introduction: Graves' disease (GD) is associated with primary hyperthyroidism, leading to weight loss before treatment. During the treatment, weight gain is frequently observed, often surpassing the initial weight loss. This study aimed to analyze weight fluctuations in GD patients, focusing on the subset of overweight and obese (OAO) individuals, considering the significant metabolic implications and heightened cardiovascular risk of these weight changes.

Methods: A retrospective cohort study included 122 GD patients with biochemical primary hyperthyroidism and at least 12 months of clinical follow-up after treatment for analysis. The OAO cohort comprised individuals with a body mass index (BMI) ≥25 kg/m². Data on laboratory, demographic, and weight variables were collected longitudinally.

Results: During the hyperthyroidism state, 34.4% (n=42) of patients presented with weight loss, a phenomenon linked to lower serum thyroid-stimulating hormone levels at diagnosis (p=0.010) and an extended need for anti-thyroid drug treatment (p<0.001). Following treatment, around 60% (n=73) of individuals encountered weight gain, exhibiting a higher prevalence among women (p<0.001) and those undergoing definitive treatment modalities (p=0.024). Notably, 26.2% (n=32) experienced excessive weight gain, which was correlated with higher premorbid BMI and diminished weight loss induced by hyperthyroidism (p<0.001). Within the OAO cohort, 66.7% (n=26) observed an increase in weight post-treatment, and in 28.2% (n=11), excessive weight gain was reported. Weight gain and excessive weight gain were noted in patients with higher initial BMIs.

Conclusions: This study highlights that post-treatment weight gain is common, emphasizing the need for careful weight management in GD. In OAO GD patients, the association between initial BMI and increased weight underscores potential cardiovascular risks, warranting vigilant monitoring and early intervention.

## Introduction

Graves’ disease (GD) stands out as a predominant cause of primary hyperthyroidism, accounting for 80% of all cases and exhibiting a wide range of clinical presentations. Notably, body mass index (BMI) fluctuations are commonly observed in these patients and are often associated with a significant clinical impact [1-3]. In individuals with hyperthyroidism, weight loss manifests through various factors, including increased energy expenditure, diarrhea, and catabolism, leading to the depletion of body fat and the loss of lean body mass [4]. A noteworthy finding from a prior study is that this weight loss is undoubtedly associated with hyperthyroidism status, regardless of an individual's premorbid weight [5].

Conversely, during GD treatment, weight gain is commonly observed. Proposed mechanisms include a reduction in thyroid hormone concentrations, potentially resulting in a decrease in resting energy expenditure [5-7]. Alton and O’Malley further speculated that hyperthyroidism might induce long-term and potentially irreversible appetite stimulation, contributing to sustained weight gain [5]. Notably, excessive weight gain, surpassing the initial weight loss, has been documented in some patients after hyperthyroidism treatment.

Considering its frequency, weight gain should be recognized as a potential complication of hyperthyroidism treatment. This holds importance in clinical practice, especially when dealing with overweight or obese individuals. The evidence clearly documents a profound association of elevated BMI with hypertension, hypercholesterolemia, and type 2 diabetes mellitus, thereby increasing patients’ cardiovascular risk [8-10]. Considering the already heightened cardiovascular risk associated with GD, effective weight control becomes imperative in order to mitigate the high morbidity and mortality risks linked to this endocrine condition [1,8].

This study aims to analyze changes in body weight among GD patients before and after treatment. Additionally, we sought to sub-analyze weight variability during treatment, specifically focusing on patients with a BMI exceeding 25 kg/m^2^. Our findings aim to equip physicians with valuable insights to accurately assess the risk of weight gain during treatment and enable proactive intervention through early recognition of patients at high cardiovascular risk.

## Materials and methods

We conducted a retrospective cohort study at the Department of Endocrinology of a tertiary referral endocrinological center, Centro Hospitalar Lisboa Ocidental, in Lisbon. The study received approval from the Ethics Committee of Hospital de Egas Moniz, Centro Hospitalar Lisboa Ocidental (approval number: 2022_EO_04). Data was collected from patients enrolled between January 2008 and January 2022 using the electronic patient database.

All patients were eligible for inclusion if they met the following criteria: age above 18 years; diagnosis of GD; a minimum follow-up period of at least 12 months after GD treatment (pharmacological or definitive treatment with surgery or radioactive iodine therapy (RAI)); and weight measurements at the premorbid state (prior to the diagnosis of hyperthyroidism), at the hyperthyroidism state (at the initial endocrinology visit with GD diagnosis), and at the post-treatment state (when biochemical euthyroidism was restored). The diagnosis of GD was established based on clinical signs of hyperthyroidism, serum thyroid-stimulating hormone (TSH) receptor antibodies (TRAbs) levels above 1.58 U/L, and TSH levels below 0.4 µUI/mL [3,11]. Patients with relapsing GD, those with treatment before the first endocrinology visit, pregnant women, and patients with thyroid malignancy were excluded.

Demographic, clinical, laboratory, and treatment data were systematically collected at specific time points: the premorbid state, the hyperthyroidism state, and the post-treatment state.

The patient’s premorbid weight was obtained from previous medical records (mainly from general practitioners) before the diagnosis of GD. Body weight at the first visit and during clinical follow-up was measured using the department’s calibrated scale. Body weight after treatment was defined as the body weight measurement when euthyroidism was obtained for the first time with treatment (regardless of treatment modality). Additionally, serum levels of TSH, free thyroxine (FT4), free triiodothyronine (FT3), TRAbs, and anti-thyroid peroxidase antibodies (TPOAbs) were recorded at the patient’s initial consultation and during treatment. Treatment modalities included anti-thyroid drugs (ATD) - methimazole or propylthiouracil - and RAI or total thyroidectomy (TT) in cases of disease relapse and persistence. The decision about the patient’s tailored therapy and its posology was determined based on physician experience, clinical characteristics, and patient preferences.

Weight loss with GD hyperthyroidism was assumed if the initial weight upon presentation to the endocrinology outpatient clinics was lower than their pre-diagnosis baseline. Moreover, weight gain with treatment was described as patients attained euthyroidism, resulting in increased weight compared to their initial measurement at the endocrinology outpatient clinics. Excessive weight gain with treatment was described as weight gain above the referred premorbid usual weight.

Remission of GD, in line with American Thyroid Association recommendations, was defined as the absence of clinical and biochemical signs of hyperthyroidism (including normal TRAbs levels) until 12 months after ATD withdrawal [12]. A specific cohort consisting of patients with BMI ≥25 kg/m² was also defined (the overweight and obese (OAO) cohort) for a specific sub-analysis of weight changes across the course of GD, using the same determined states described previously.

Thyroid function assessments

Thyroid function tests were measured using an automated direct chemiluminescent method (normal range: TSH 0.4 to 4.2 μIU/mL, FT4 12.0 to 22.0 pmol/L, and FT3 3.10 to 6.80 pmol/L). Thyroid autoantibody assays were also performed using an automated chemiluminescent method (normal range: TRAbs 0 to 1.58 U/L and TPOAbs <35 UI/mL).

Statistical analyses

All Gaussian distributed variables were expressed as mean (standard deviation (SD)) and nonnormally distributed variables as median (interquartile range (IQR)). Continuous variables were expressed as numbers and percentages. The chi-square test was used for categorical variables, and the t-test and Kruskal-Wallis were used on continuous variables for statistical assessment of outcomes between groups.

In all the hypothesis tests, a p-value of less than 0.05 was considered for statistical significance, and the usual confidence intervals of 95% were chosen. Missing values were handled by patients’ exclusion if they had a missing rate higher than 5% of the analyzed variables in order to eliminate any associated missing at random bias. All remaining missing values were treated as missing at random, and univariate feature imputation with median values was used. All statistical analyses were performed using SPSS Statistics version 21 (IBM Corp. Released 2012. IBM SPSS Statistics for Windows, Version 21.0. Armonk, NY: IBM Corp.).

## Results

**Figure 1:**
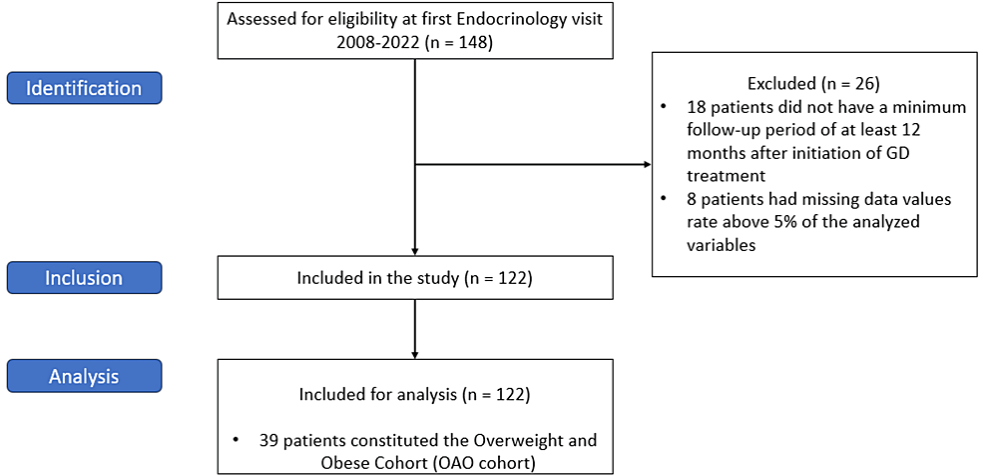
Patients’ selection for the analysis using the STROBE statement STROBE: strengthening the reporting of observational studies in epidemiology, GD: Graves' disease, OAO: overweight and obese

One hundred and forty-eight patients were initially eligible for the study. Of these patients, 26 were excluded from the statistical analysis, 18 because they did not have a minimum follow-up period of at least 12 months after initiation of GD treatment, and eight patients due to missing data values above 5% of the analyzed variables. The remaining 122 patients were included, as depicted in Figure 1.

The included patients for statistical analysis were predominantly females (95 individuals), with a mean age of 48 years (±15). The patients were followed up for an average duration of 2.8 years (IQR 1.3-4.6) with regular visits to the endocrinology outpatient clinics.

During the course of the GD hyperthyroidism state, weight reduction was observed in approximately 34.4% (n=42) of patients, with an average loss of 5.7 kg (±8.0) and a mean decrease in BMI of 2.1 kg/m² (±3.1). Notably, patients with weight loss presented with lower serum levels of TSH (0.001 (0.001-0.010) vs. 0.010 (0.001-0.150), p=0.01) without differences in baseline TRAb serum levels when compared to patients without weight loss. Patients experiencing weight loss further required a longer duration of ATD (24.1 ± 15.7 vs. 17.4 ± 12.9, p=0.013) to achieve disease remission (Table 1).

**Table 1:** Demographic and laboratory characteristics in patients with and without weight loss during the hyperthyroidism state TSH: thyroid-stimulating hormone serum levels, fT4: free thyroxine serum levels, fT3: free triiodothyronine serum levels, TRAb: TSH receptor antibodies serum levels, TPOAb: thyroid peroxidase antibodies serum levels, ATD: anti-thyroid drug

	Patients with weight loss during hyperthyroidism state (n=42; 34.4%)	Patients without weight loss during hyperthyroidism state (n=80; 65.6%)	p-value
Age (years), median (IQR)	51.6 (39.1-62.1)	43.4 (38.6-52.2)	0.400
Premorbid BMI (kg/m^2^), median (IQR)	22.1 (19.3-24.2)	24.8 (21.7-29.0)	0.859
Baseline TSH (uUI/mL), median (IQR)	0.001 (0.001-0.010)	0.010 (0.001-0.150)	0.010
Baseline fT4 (pmol/L), median (IQR)	50.0 (38.3-60.9)	30.0 (22.8-53.7)	0.071
Baseline fT3 (pmol/L), median (IQR)	15.1 (8.2-19.9)	12.9 (6.8-17.2)	0.770
Baseline TRAb (U/L), median (IQR)	6.4 (2.7-16.0)	5.1 (2.7-9.6)	0.133
TPOAb (U/L), median (IQR)	250 (59.0-620.0)	1000 (200.0-1300)	0.091
ATD treatment duration (months), mean (sd)	24.1 (±15.7)	17.4 (±12.9)	0.012

All GD patients underwent initial medical intervention with ATD agents for an average duration of 18 months (IQR 14-24), with a remission rate of 51.6% (n=63). Methimazole was the primary therapeutic choice, selected as the first-line therapy in the majority of cases (n=97, 79.5%). In cases where patients were not responsive to medical therapy, had uncontrolled symptoms, or required prompt euthyroidism restitution, a TT (n=19) or an RAI (n=8) was performed.

After euthyroidism was restored using ATD or TT/RAU, 59.8% (n=73) of patients experienced an increase in body weight compared to the corresponding morbid state. Median weight and BMI gain were 2.3 kg (IQR 0.03-9.00) and 1.9 kg/m² (±2.7), respectively. Weight gain was more common in women (p<0.001) and more pronounced in patients submitted to definitive treatment modalities (either by TT or RAI) compared to those treated only with medical treatment (BMI +3.1 (0.9-6.6) vs. +0.9 ((-0.2)-3.2) kg/m², p=0.024). Weight gain was more prevalent after TT compared to RAI (p<0.001), with all TT patients presenting with weight gain.

Additionally, 26.2% (n=32) of all patients experienced excessive weight gain after treatment (Table 2). Patients experiencing this excessive weight gain presented with a higher BMI in the premorbid state (p=0.002) and had a significantly lower occurrence of weight loss during the hyperthyroidism state (p<0.001).

**Table 2:** Demographic and laboratory characteristics and BMI variation profiles in patients with and without excessive weight gain after GD treatment TSH: thyroid-stimulating hormone serum levels, fT4: free thyroxine serum levels, fT3: free triiodothyronine serum levels, TRAb: TSH receptor antibodies serum levels, TPOAb: thyroid peroxidase antibodies serum levels, BMI: body mass index, GD: Graves’ disease, ATD: anti-thyroid drug

	Patients with excessive weight gain after GD treatment* (n=32, 26.2%)	Patients without excessive weight gain after GD treatment* (n=90, 73.8%)	p-value
Age (years), median (IQR)	51.8 (40.8-66.0)	56.9 (44.6-62.6)	0.014
Baseline TSH (uUI/mL), median (IQR)	0.010 (0.003-0.019)	0.010 (0.001-0.011)	0.119
Baseline fT4 (pmol/L), median (IQR)	34.6 (27.8-45.4)	54.8 (28.9-62.8)	0.607
Baseline fT3 (pmol/L), median (IQR)	14.3 (9.4-18.0)	13.4 (8.4-17.2)	0.587
Baseline TRAb (U/L), median (IQR)	4.3 (2.0-11.0)	11.6 (2.5-27.3)	0.175
TPOAb (U/L), median (IQR)	1000.0 (100.0-1300.0)	935.0 (457.3-1300.0)	0.266
Premorbid BMI (kg/m^2^), median (IQR)	29.4 (27.0-30.8)	22.5 (25.6-28.1)	0.002
BMI variation in hyperthyroidism state (kg), median (IQR)	- 0.0 ((-1.6)-0)	-2.3 ((-4.1)-(-0.4))	0.020
ATD treatment duration (months), mean (SD)	13.0 (11.3-17.3)	10.0 (6.8-15)	0.006

The OAO subgroup constituted 31.9% (n=39) of the patient population, characterized by a BMI ≥25 kg/m². No differences were found in the biochemical GD markers and BMI dynamics between the OAO cohort and the non-OAO cohort (Table 3).

**Table 3:** Demographic, laboratory characteristics, and BMI variation profiles between patients from the OAO cohort and the non-OAO cohort TSH: thyroid-stimulating hormone serum levels, fT4: free thyroxine serum levels, fT3: free triiodothyronine serum levels, TRAb: TSH receptor antibodies serum levels, TPOAb: thyroid peroxidase antibodies serum levels, TGAb: thyroglobulin antibodies serum level, BMI: body mass index, OAO: overweight and obese

	OAO (n=39, 31.9%)	Non-OAO (n=83; 68.1%)	p-value
Baseline TSH (uUI/mL), median (IQR)	0.011 (0.001-0.01)	0.001 (0.001-0.010)	0.534
Baseline fT4 (pmol/L), median (IQR)	40.8 (27.1-60.3)	46.2 (33.1-60.5)	0.486
Baseline fT3 (pmol/L), median (IQR)	12.8 (8.2-15.8)	16.6 (6.1-25.7)	0.415
Baseline TRAb (U/L), median (IQR)	6.9 (3.0-15.3)	7.6 (2.7-18.5)	0.874
TPOAb (U/L), median (IQR)	620.0 (100.0-1000.0)	850.0 (51.8-1000.0)	0.819
TGAb (U/L), median (IQR)	505.0 (168.5-961.0)	225.0 (51.8-907.0)	0.226
BMI variation in patients with weight gain after treatment (kg/m^2^), median (IQR)	+0.92 (-0.44-4.38)	+1.50 (0.33-3.38)	0.605
BMI variation in patients with excessive weight gain after treatment (kg/m^2^), median (IQR)	0.00 (-10.05-4.10)	+0.850 (-1.64-2.36)	0.088

This cohort consisted of 28 women (71.8%), with a mean age of 49.1 (±15.5) years. The majority of patients in this subgroup were classified as overweight (82.1%, n=32), while six individuals demonstrated grade 1 obesity and one patient presented with grade 2 obesity. Approximately two-thirds of these patients (66.7%, n=26) showed an elevation of weight and BMI after treatment (BMI at the premorbid and post-treatment status of 27.1 ± 3.8 vs. 29.2 ± 5.2). Among those, weight gain was more prevalent in older individuals (p=0.014), females (p=0.015), and those undergoing a prolonged course of ATD treatment (p=0.006). Excessive weight gain was observed in 28.2% (n=11) of the OAO patients. Moreover, weight gain and excessive weight gain were observed in OAO patients with higher initial BMIs (29.7 ± 3.4 vs. 26.1 ± 0.83 and 28.1 ± 2.8 vs. 25.7 ± 2.6, p=0.024 and p=0.03, respectively).

## Discussion

Our results clearly show significant weight fluctuations in GD patients, influenced by different pathological states and therapeutic options, highlighting the intricate interplay between thyroid hormones and energy intake/expenditure. It is already well established that, due to hyperthyroidism, GD patients typically experience weight loss before treatment initiation [1-3,12]. Consistent with these findings, our results clearly depict that a significant 34.4% (n=42) of patients exhibited a weight reduction during the course of the GD hyperthyroidism state. Patients with this weight behavior presented lower values of TSH at the diagnosis and required prolonged exposure to AT, suggesting that a more severe disease may be associated with more pronounced weight loss.

However, compelling evidence indicates that post-treatment weight gain, albeit common, is not easily identified and can sometimes even exceed premorbid levels [5,13]. In our study, although only one-third of patients exhibited significant weight loss during the hyperthyroid state, the majority of patients (59.8%, n=73) experienced weight gain, with 26.2% (n=32) registering excessive weight gain during treatment. Some authors have hypothesized that a substantial portion of the initial weight gain after hyperthyroidism therapy consists of an increase in muscle mass (lean body mass) [4,13,14]. On the other hand, previous studies have suggested that the characteristic increased appetite in these patients, secondary to hyperthyroidism, is compensatory for the initial state of negative energy balance and might persist chronically, even after the restoration of euthyroidism [5,15]. Regardless of the mechanism, our results indicate a higher prevalence of weight gain with GD treatment than reported in previous studies, highlighting the risk of further metabolic challenges due to an increase in weight in these patients [16].

Our findings additionally illustrate that weight gain in the post-therapeutic state was more commonly observed in women and individuals undergoing definitive treatment modalities (either by TT or RAI) compared to patients receiving only medical treatment. These results denote that certain populations may be associated with a higher susceptibility to weight gain, emphasizing that determined clinical factors should be taken into consideration in daily clinical practice. This insight enables the tailoring of directed therapies to specific at-risk patient groups, particularly in the context of high cardiovascular-risk patients [17-19].

Our data on the OAO cohort may further reinforce the need for weight control measures in treated GD patients, particularly those with previous excessive weight who are at high risk of metabolic syndrome. Our results show an increase in weight in 66.7% (n=26) of those patients, particularly in older subjects with a higher premorbid BMI. Furthermore, 28.2% (n=11) of OAO patients exhibited excessive weight gain. These results are particularly worrisome and may prompt true concerns when considering the heightened cardiovascular risk in GD patients [8,19-21]. This aligns with another study demonstrating a significant increase in OAO status in a pediatric population after RAI treatment, placing them at higher cardiovascular risk [22]. In the future, research on the rate of cardiovascular events and morbidity associated with these weight fluctuations in GD patients will be of paramount relevance to preventing potential future complications, cardiovascular morbidity, and mortality.

Our study has several strengths. To the best of our knowledge, this is one of the largest cohorts evaluating weight changes in GD patients in the existing medical literature with a reasonable time of follow-up. It also explores the biochemical and endocrinological characteristics of these patients, allowing for the consideration of disease severity in these weight fluctuations. Additionally, it has a minimum drop-out rate and a negligible rate of missing data imputation (<1%), avoiding potential bias.

However, we acknowledge some limitations in our study. It is retrospective in nature and conceptually retrieves the patient’s premorbid weight from previous medical records before the evaluation of the patient in the endocrinology outpatient clinic. Furthermore, potential confounding factors such as patients' baseline health status, socioeconomic status, dietary habits, physical activity levels, and other medications were not considered. Additionally, the independent evaluation and therapeutic decision of the attending physician were used as key elements for GD therapeutic planning and management in different disease states. Nonetheless, this approach also reflects real-world conditions faced by physicians in endocrinological tertiary referral centers managing GD patients.

## Conclusions

Our study meticulously documents significant weight fluctuations influenced by disease status and therapeutic interventions, emphasizing the intricate interplay between thyroid hormones and energy balance. These results underscore the imperative to establish weight control measures in GD patients, extending beyond the regulation of thyroid hormone levels. This is especially crucial for patients with obesity and overweight, as they exhibit a higher risk for metabolic syndrome and cardiovascular morbidity and mortality.

Weight management should be a focal point for all patients undergoing treatment for GD hyperthyroidism. Comprehensive strategies, including dietary, lifestyle, and pharmacological interventions, should be implemented to minimize the development of obesity and alleviate the detrimental effects of weight gain in this population.
